# The prevalence of effort-reward imbalance and its associations with working conditions, psychosocial resources and burden among health care workers during the COVID-19 pandemic: Results of the egePan-Voice study

**DOI:** 10.1371/journal.pone.0287509

**Published:** 2023-08-17

**Authors:** Petra Beschoner, Lucia Jerg-Bretzke, Yesim Erim, Franziska Geiser, Andreas M. Baranowski, Kerstin Weidner, Christian Albus, Caterina Schug, Kerstin Limbrecht-Ecklundt, Katja Weimer, Marc N. Jarczok, Maximilian Kempf, Harald Gündel, Eva Morawa

**Affiliations:** 1 Department of Psychosomatic Medicine and Psychotherapy, Ulm University Medical Center, Ulm, Germany; 2 Department of Psychosomatic Medicine and Psychotherapy, University Hospital of Erlangen, Friedrich-Alexander University Erlangen-Nürnberg (FAU), Erlangen, Germany; 3 Department of Psychosomatic Medicine and Psychotherapy, University Clinic of Bonn, Bonn, Germany; 4 Department of Psychotherapy and Psychosomatic Medicine, Carl Gustav Carus Faculty of Medicine, Technische Universität Dresden, Dresden, Germany; 5 Department of Psychosomatics and Psychotherapy, Medical Faculty and University Hospital, University of Cologne, Cologne, Germany; State Health Resource Center, Chhattisgarh, INDIA

## Abstract

**Objective:**

The association between a measure of effort-reward imbalance (ERI) and profession as well as gender in a sample of health care workers (HCW) during the first wave of the COVID-19 pandemic in Germany using the egePan-Voice study. In addition, we examined, which factors are associated with an effort-reward imbalance ratio (ERI ratio) >1.

**Methods:**

In a large sample of HCW (N = 6174) we assessed occupational stress with the short version of the effort-reward imbalance (ERI) questionnaire, working conditions, COVID-19-related problems and psychosocial resources (ENRICHD Social Support Inventory, ESSI; Sense of Coherence Scale, SOC-3 and optimism, SOP2).

**Results:**

The prevalence of a ERI ratio >1 among HCW was 50.9%. The prevalence’s of an ERI ratio >1 were statistically significant different between gender as well as the occupational profession. The proportion of women (51.8%) with ERI ratio >1 was significantly higher than among men (47.8%). The highest ERI imbalance was found among nurses (62.8%), followed by medical technical assistants (MTA) (58.8%), while psychologists/psychotherapists revealed the lowest value (37.8%), followed by physicians (41.8%). In the total sample, most essential factors reported at this time for increased ERI ratio were: insufficient staff for the current work load, insufficient recovery, feeling insufficiently protected by measures taken by the hospital/the employer, high occupancy rate of the wards, insufficient trust in colleagues and being a nurse as compared with being a physician.

**Conclusion:**

The findings indicate a high proportion of HCW with effort-reward imbalance and substantial profession-related differences. Preventive interventions should be offered to vulnerable groups among the HCW to decrease the imbalance measured by work stress.

## Introduction

Pandemics are associated with high mental stress among health care workers (HCW) [1, 2] who are at the forefront of treating patients with a life-threatening illness. Besides the increase in workload to the breaking point, this often leads to confrontation with great suffering, dying and death, some have to make difficult decisions especially in triage situations [3]. A study with more than 3600 German HCW showed that HCW who worked in a COVID-19 environment reported higher levels of subjective burden and stress compared to all other participants [4].

Recent international studies also show high levels of stress and psychological symptoms in these professions for the current COVID-19 pandemic (for an overview, see [2, 5–7]). Occupational stress and mental health of HCW affect the quality of patient care [8], and is thus essential for coping with a pandemic.

The majority of studies available to date on medical staff stress from the COVID-19 pandemic are from the Asian region and cover mainly nurses and physicians. Some studies also examined other medical professional groups [9, 10]. However, in these studies were no decided distinctions made between the occupational groups. Two recent studies reported high general stress levels among hospital staff [11] and high stress levels among medical and non-medical hospital staff, respectively [12]. Findings from a Spanish study showed lower emotional impact among administrative and medical staff compared to nurse aides and medical assistants [13]. Another European cross-sectional study showed significantly higher scores of depression and anxiety in nonmedical professionals [14]. Kramer et al. studied over 3600 HCW in Germany and found higher occupational stress in nurses compared to physicians and other staff [4].

Therefore, it is important to identify which specific factors of a pandemic may promote or trigger occupational stress in different health care professions. At the same time, resources must be identified that can protect against this stress and the possible mental symptoms that may follow.

A well-established model in international research for the emergence and recording of occupational stress is Siegrist’s effort-reward imbalance model [15, 16]. It describes the development of psychosocial work stress through an imbalance between work effort (e.g., demands, obligations) and received reward (e.g., wages, recognition, job security) at work. If there is an effort-reward imbalance to the disadvantage of recognition, a so-called effort-reward imbalance, there is an empirically well-documented risk of developing psychological and physical stress symptoms and even manifest illnesses [17]. This relationship has been well studied in different occupational groups [18–22].

It is likely that psychosocial workload (effort-reward imbalance) also plays a role in the stress experience of healthcare professionals in the COVID-19 pandemic, as demands have continued to increase or change in many areas. For example, correlative associations between workload (effort) and depressive symptoms were shown among anesthesiologists during the COVID-19 pandemic [23]. A Chinese study of HCW also presented correlations between work effort, depressive symptoms, and anxiety symptoms during the pandemic [24].

Well-studied, established concepts on protective variables and resources are optimism, social support, and sense of coherence [25, 26].

The term social support encompasses various concepts, whereby the concept of perceived social support is considered particularly helpful. This is about the experienced availability of support. It is well established that this concept is related to the experience of stress [26]. In the COVID-19 pandemic, perceived social support was also found to be a protective factor with regard to stress experience in both the general population [27] and HCW [28].

Sense of coherence (SOC) represents a resilience concept that encompasses how much individuals experience their world as comprehensible, manageable, and meaningful. SOC is considered an important predictor of mental health in the general population [25]. A higher SOC is also associated with fewer mental health problems in HCW [29–31].

Optimism is considered a relatively stable personality trait [32], reflecting the extent to which a person has general positive expectations for the future [33]. Optimism is positively associated with mental and physical well-being, life satisfaction and job satisfaction [34–36]. And it is negatively associated with depression, suicidality, and feelings of helplessness [35, 37, 38]. Studies on HCW show a positive influence of optimism on quality of life as well as a negative association of optimism with burnout [39, 40]. Studies during the COVID-19 pandemic confirmed positive effects of optimism on stress and mental symptoms in HCW [41, 42].

As work stress in HCW is related to optimism, sense of coherence and social support have not been the focus of research on the COVID-19 pandemic, this study aims to clarify their role in the context of HCWs particular work stresses in the COVID-19 pandemic.

From this we concluded the following aims for the present study:

1. To determine the level of job-demands (ERI-effort) and reward (ERI-reward) and the prevalence of effort-reward imbalance ratio (ERI ratio >1) among HCW during the first wave of the COVID-19 pandemic in dependence of gender and profession.
2. To analyse which factors (sociodemographic, occupational, COVID-related, working conditions during the COVID-19 pandemic, psychosocial resources and change in distress) are associated with efforts, rewards, and the effort-reward imbalance ratio (ERI ratio >1) (for the total sample and stratified for gender).

Due to the explorative nature of both research aims we do not formulate hypotheses.

Based on these aims, we expect to be able to identify fields of action to support HCW in future epidemics in the best possible way and thus keep them healthy and efficient.

## Method

### Statement of ethics

The present study was approved by the Ethics Committee of the Medical Faculty of the Friedrich-Alexander University Erlangen-Nürnberg (FAU) and registered in the German Clinical Trials Register (DRKS-ID: DRKS00021268). All respondents provided their online informed consent by actively clicking a button on the particular online survey tool prior to data collection.

### Data collection

A web-based survey consisting of 77 items (approximately 15 min. filling time) was conducted between April 20 and July 5, 2020 by the psychosomatic departments of the university hospitals of Erlangen, Bonn, Ulm, Cologne, and Dresden. This was the first measurement of the prospective VOICE study being a part of the egePan Unimed project (Development, testing and implementation of regionally adaptive care structures and processes for evidence-based pandemic management coordinated by University Medical Center) that is supported by the German Federal Ministry of Education and Research (BMBF). The survey was shared via online platforms or mailing lists. Further hospitals and various professional associations and online platforms promoted participation in the survey. Follow-up measurements were promoted analogously. By generating a pseudonymization code in the further measurements, conditions were created for different analysis possibilities for the longitudinal data. Thus, it is possible to view the cross-sectional data separately. The results can be viewed as in a trend study or, if the number of cases is sufficient, they can be treated as a panel study.

The survey was provided via two academic online survey tools, Unipark (www.unipark.com) and SoSci Survey (www.soscisurvey.de). After being informed about the content of the survey, respondents explicitly confirmed their voluntary participation and further data processing by clicking a button. Inclusion criteria were a minimum age of 18 years, working in the health care sector, residence/working place in Germany, and sufficient German language skills.

### Response rate and gender proportion

Due to heterogeneous recruitment strategies, the response rate for the total sample could not be measured. The only directly addressed target group was the four university hospitals. The response rates of the largest occupational groups over the total sample were: 10.0% (8.1–13.3) physicians, 8.9% (5.9–10.1) nurses and 24,5% (13,8–37,4) MTA. Three quarters of the total sample was female [43].

## Measures

### Sociodemographic and occupational characteristics

The following sociodemographic data were assessed: age (*age categories*: *18–30*, *31–40*, *41–50*, *51–60*, *61–70 and >70 years*), gender (*men*, *women*, *diverse*), living alone (*or not*), having children (*in the household* or *not in the household* or *not having children*), care for sick/old relatives (*in the household* or *not in the household*) and migration background (if the respondent or at least one parent did not have the German citizenship by birth, Federal Statistical Office, 2020).

Assessed occupational characteristics were employment status, working in patient care, the working setting, and profession.

The following COVID-19-related variables were assessed: change of the department due to the pandemic, having direct contact at work with COVID-19 infected patients proved by a test, contact with contaminated material during work, presently working in home office, degree of occupancy of the wards, belonging to an at-risk group because of a preexisting illness.

### Working conditions during the COVID-19 pandemic

Working conditions during the Covid-19 pandemic were measured with the following items on a scale from 0 “strongly disagree”to 4 “strongly agree”(with regard to the last two weeks): “There is sufficient staff for the current work load.”; “I can recover sufficiently during my free time.”; and “I can trust in my colleagues, when it gets difficult during work.”

### Potential problems in the COVID-19 pandemic

The following potential problems in the COVID-19 pandemic were assessed with a Likert scale from 0 “strongly disagree”to 4 “strongly agree”(with regard to the last two weeks): “I was afraid to become infected”; “I was afraid to infect relatives or my family.”; “I felt protected by measures taken by national and local authorities.”; “I felt protected by measures taken by the hospital/ the employer.”. These items were derived from a former study [44].

### Change in distress

The overall burden level during and (retrospectively) before the COVID-19 pandemic was measured with a single item, respectively. Participants were asked “How much burden have you felt due to the COVID-19 pandemic in the last 2 weeks?” and “How much burden did you feel before the COVID-19 pandemic?” The scale ranged from 0 "not at all" to 4 "very strong". To determine the increase in subjective burden levels we calculated a difference score between these two items (during the pandemic–before the pandemic). Higher values reflect a higher increase in subjective burden due to the COVID-19 pandemic.

### Effort-reward imbalance

Work stress was assessed with the short version of the effort-reward imbalance questionnaire (ERI, [17]) comprising ten items (three for the effort scale and seven for the reward scale) on a five-point Likert scale. Higher ratings indicate higher efforts and rewards, respectively. The effort-reward ratio was calculated through dividing the sum score of the effort scale with the sum score of the reward scale, adjusted for the unequal amount of items. ERI ratio >1 indicate substantial stress at work. Cronbach´s Alpha in the present study was 0.74 for both the effort scale and the reward scale.

### Social support

Perceived emotional social support was measured with the German version of the ENRICHD Social Support Inventory (ESSI) that consists of five items on a five-point Likert scale (1 = never to 5 = always) [45]. The sum score ranges from 5 to 25. Higher scores reflect higher levels of perceived emotional social support. A cut-off value of ≤ 18 and the answer of at least two items ≤ 3 indicates low social support. The internal consistency (Cronbach´s Alpha) in the present study was 0.90.

### Sense of coherence

Sense of coherence was assessed with the German ultrashort version (SOC-3) of the Sense of Coherence Scale [46] comprising three items [47]. It is based on Antonovsky´s original 29 item questionnaire. The sum score for the SOC-3 ranges from 3 to 21. Higher values indicate a stronger SOC. Cronbach´s Alpha in the present study was 0.70.

### Optimism

Optimism was assessed with the item “How optimistic are you in general?” [48] with a seven-point Likert scale from 1 = “not optimistic at all” to 7 = “very optimistic”.

The survey also included questionnaires measuring e.g. depressive, anxiety and post-traumatic symptoms and work family conflict. The results for these questionnaires will be analyzed in other publications.

### Statistical analysis

SPSS V. 28 was used for data analyses. Missing values in the total sample (N = 6174) were identified in 5.5% of the participants for the ERI, in 2.3% for each of the ESSI items, in 2.7% in each of the SOC-3 items, in 2.9% of the optimism item and in 0.8% for one item concerning working conditions during the COVID-19 pandemic. Missing data were imputed using the expectation-maximization algorithm. The assumption that missing values are missing completely at random was examined by the Little´s MCAR test. Descriptive statistics (absolute and relative frequencies) were computed to describe the sociodemographic and occupational characteristics of the study sample. The assumption of normality was checked by visual inspection (histograms, Q-Q plots and boxplots). Group differences were tested with the χ^2^-test and the Z-test for categorical variables and the t-test for independent samples or the Welch test (because of heterogeneity of variances and unequal sample sizes), with Games-Howell adjusted post hoc analysis for continuous variables, where appropriate. The effect sizes (Cohen´s d, Phi, Cramer´s V and partial Eta^2^) are also reported (d ≥ 0.2 = small, d ≥ 0.5 = medium and d ≥ 0.8 = large effect size; φ/V ≥ 0.1 = small, φ/V ≥ 0.3 = medium and φ/V ≥ 0.5 = large effect size; η^2^_p_ ≥ 0.01 = small, η^2^_p_ ≥ 0.06 = medium, η^2^_p_ ≥ 0.14 = large effect size) (Cohen, 1988). Pearson correlation analyses were conducted to explore the relationship between ERI-effort and ERI-reward and the assessment of working conditions, potential COVID-related problems, psychosocial resources and change in distress. Multiple linear regression analyses and Odds Ratios were calculated to examine the associations between sociodemographic, occupational, and COVID-related variables, working conditions during the COVID-19 pandemic, psychosocial resources and change in distress with the effort-reward imbalance ratio for the total sample and stratified for gender. To detect multicollinearity, variance inflation factors (VIF) were computed. A level of significance of p < .05 (two-tailed) was predetermined in all analyses except for the case of alpha error correction (then explicitly reported in the text).

## Results

The total sample consisted of N = 8061 health care professionals (T1). In this paper, we focused on the ERI as the main variable of interest. We included for analyses only respondents with max. 1 missing value in the ERI questionnaire. Thus, the total sample for the present manuscript comprises N = 6174 HCW.

### Sociodemographic and occupational variables

Data on sociodemographic and occupational characteristics of the study sample are presented in Table 1. Two fifths of the sample (41.2%) were younger than 41 years. A large proportion of the sample were working in fulltime (61%), patient care (83.3%) and in a university hospital (38.5%). Physicians (25.6%) and MTA (24.7%) were the most common professions among the respondents, followed by non-medical health professions/ pedagogues (15.0%), nurses (14.9%), psychologists/ psychotherapists (6.0%) and administration staff (5.6%). Other occupational groups accounted for 8.2%.

**Table 1:** Socio-demographic and occupational characteristics of the study sample* and frequency of effort-reward imbalance ratio (ERI ratio >1) (N = 6174).

	Total sample N = 6174	ERI>1 n (%)	p value (Phi/Cramer´s V)
**Gender. n (%)**			**0.008 (0.034)+**
Women	4764 (77.2)	2468 (51.8)	
Men	1396 (22.6)	667 (47.8)	
Divers	14 (0.2)	5 (35.7)	
**Age. years. n (%)**			**0.026 (0.042)**
18–30	1146 (18.6)	579 (50.5)	
31–40	1395 (22.6)	735 (52.7)	
41–50	1478 (23.9)	764 (51.7)	
51–60	1736 (28.1)	879 (50.6)	
>60	419 (6.8)	183 (43.7)	
**Living alone. n (%)**			**0.004 (0.037)**
Yes	1328 (21.5)	722 (54.4)	
No	4846 (78.5)	2418 (49.9)	
**Children. n (%)**			**0.007 (0.034)**
Yes	3483 (56.4)	1719 (49.4)	
No	2691 (43.6)	1421 (52.8)	
**Care for sick/old relatives. n (%)**			**<0.001 (0.050)**
Yes	1117 (18.1)	628 (56.2)	
No	5057 (81.9)	2512 (49.7)	
**Migration background. n (%)**			0.905 (0.002)
Yes	648 (10.5)	331 (51.1)	
No	5526 (89.5)	2809 (50.8)	
**Employment. n (%)**			**<0.001 (0.058)**
Full-time	3764 (61.0)	2002 (53.2)	
Part-time	2410 (39.0)	1138 (47.2)	
**Working in patient care. n (%)**			0.689 (0.005)
Yes	5142 (83.3)	2621 (51.0)	
No	1032 (16.7)	519 (50.3)	
**Working setting. n (%)**			**<0.001 (0.095)**
University hospital	2375 (38.5)	1233 (51.9)	
Non-university hospital	1221 (19.8)	696 (57.0)	
Private practice	684 (11.1)	271 (39.6)	
Social pediatric	1203 (19.5)	607 (50.5)	
Other	691 (11.2)	333 (48.2)	

*The group consisted of the following professional subgroups: Medical assistants. Medical-technical laboratory assistants. Medical-technical radiology assistants. Pharmaceutical-technical assistants; +only for women and men; significant p values with corresponding effect sizes are marked in bold.

### COVID-19-related characteristics, problems, and working conditions

Almost two fifths of the sample had contact with infected patients (37.6%) or with contaminated material (38.4%) at work (Table 2). One in seven HCW (14.1%) had to change the department due to the COVID-19 pandemic and 22.2% were working at home office exclusively or partly. The occupation rate of the wards was assessed as slightly or strongly above average by one quarter of the participants (25.5%). Almost twice as much HCW demonstrated fear to infect family with COVID-19 (49.7%) than to infect oneself (26.5%). Approximately half of the participants (47.8%) felt protected by measures of the hospital/ the employer, while 39.6% felt protected by measures of national/ local authorities. Concerning the working conditions, a considerable proportion rated the amount of the personnel for the current work load (46.0%) and the recovery in free time (60.0%) as insufficient. Trust in colleagues, when it gets difficult during work was reported by approx. 70%.

**Table 2:** COVID-19-related characteristics and problems and working conditions of the sample and frequency of effort-reward imbalance ratio (ERI ratio >1) (N = 6174).

	Total sample N = 6174	ERI>1 n (%)	p-value (Phi/ Cramer´s V)
**Contact with infected patients. n (%)**			**<0.001 (0.080)**
Yes	2319 (37.6)	1299 (56.0)	
No	3855 (62.4)	1841 (47.8)	
**Contact with contaminated material. n (%)**			**<0.001 (0.136)**
Yes	2370 (38.4)	1409 (59.5)	
No	3804 (61.6)	1731 (45.5)	
**Risk group due to preexisting illness. n (%)**			**<0.001 (0.100)**
Yes	1203 (19.5)	734 (61.0)	
No	4971 (80.5)	2406 (48.4)	
**Change of department. n (%)**			**0.021 (0.029)**
Yes	872 (14.1)	475 (54.5)	
No	5302 (85.9)	2665 (50.3)	
**Home office. n (%)**			**<0.001 (0.059)**
Yes	1373 (22.2)	622 (45.3)	
No	4801 (77.8)	2518 (52.4)	
**Occupation rate of the wards. n (%)**			**<0.001 (0.232)**
Slightly/strongly above average	1577 (25.5)	1114 (70.6)	
Strongly/slightly below average/average	4597 (74.5)	2026 (44.1)	
Sufficient staff for the current work load*. **n (%)**			**<0.001 (0.311)**
Yes	3331 (54.0)	1216 (36.5)	
No	2843 (46.0)	1924 (67.7)	
Sufficient recovery in free time*. **n (%)**			**<0.001 (0.297)**
Yes	2471 (40.0)	807 (32.7)	
No	3703 (60.0)	2333 (63.0)	
Trust in colleagues*. **n (%)**			**<0.001 (0.228)**
Yes	4318 (69.9)	1881 (43.6)	
No	1805 (29.2)	1238 (68.6)	
Missing	51 (0.8)	-	
Fear to become infected with COVID-19*. **n (%)**			**<0.001 (0.138)**
Yes	1637 (26.5)	1021 (62.4)	
No	4537 (73.5)	2119 (46.7)	
Fear to infect family with COVID-19*. **n (%)**			**<0.001 (0.159)**
Yes	3069 (49.7)	1806 (58.8)	
No	3105 (50.3)	1334 (43.0)	
Feeling protected by measures of the national/local authority*. **n (%)**			**<0.001 (0.162)**
Yes	2444 (39.6)	998 (40.8)	
No	3730 (60.4)	2142 (57.4)	
Feeling protected by measures of the hospital/the employer*. **n (%)**			**<0.001 (0.247)**
Yes	2952 (47.8)	1121 (38.0)	
No	3222 (52.2)	2019 (62.7)	

*dichotomized: yes = strongly agree/ rather agree. no = neither agree nor disagree/ rather disagree/ strongly disagree; significant p values with corresponding effect sizes are marked in bold.

### Levels of ERI-effort and ERI-reward

No significant difference was observed between men (M = 7.60, SD = 2.24) and women (M = 7.55, SD = 2.21) with regard to the scale “effort” (t(6158) = 0.772, p = 0.440; see Table 3). In terms of the “reward” scale, men (M = 17.92, SD = 4.07) scored significantly higher than women (M = 17.28, SD = 3.75; t(2140,522) = 5.299, p<0.001), however the effect size was very small (d = 0.168; see Table 3).

**Table 3:** ERI-effort and ERI-reward levels in dependence of gender (n = 6160) and profession (n = 6174).

Gender	N	ERI-Effort, Mean (SD)	Cohen´s d	p value	ERI- reward, Mean (SD)	Cohen´s d	p value
Men	1396	7.60 (2.24)	0.023	0.440	17.92 (4.07)	**0.168**	**<0.001**
Women	4764	7.55 (2.21)			17.28 (3.75)		
**Profession**							
Physicians	1580	7.50 (2.24)	**0.016**	**<0.001**	18.80 (3.96)	**0.068**	**<0.001**
Nurses	923	8.06 (2.19)			16.41 (3.48)		
MTA	1526	7.63 (2.16)			16.35 (3.54)		
Psychologists/ Psychotherapists	370	7.02 (2.08)			18.30 (3.35)		
Non-medical health professions/Pedagogues	924	7.47 (2.10)			17.22 (3.79)		
Administration staff	346	7.74 (2.41)			17.38 (3.87)		
Others	505	7.09 (2.31)			17.92 (3.74)		

ERI = effort-reward imbalance questionnaire; MTA = medical technical assistants; significant p values with corresponding effect sizes are marked in bold.

A significant association was also detected between the profession and the level of effort (Welch´s F(6, 1784.696) = 16.76, p<0.001). Nurses (M = 8.06, SD = 2.19) reported on average significantly (p<0.001) higher score for the “effort” scale in comparison with all other occupational groups but the administration (M = 7.74, SD = 2.41, p = 0.335) (Table 3). Psychologists/psychotherapists (M = 7.02, SD = 2.08) showed significantly lower efforts as compared with all other professions.

Concerning the “reward” scale the mean value also differed statistically significantly in dependence of the profession (Welch´s F(6, 1795.387) = 74.074, p<0.001). MTA (M = 16.35, SD = 3.54) showed significantly (p<0.001) lower scores for reward in comparison with all other occupational groups except nurses (M = 16.41, SD = 3.48, p = 1.0) and nurses as compared with all other HCW but MTA (Table 3). Physicians (M = 18.80, SD = 3.96) revealed significantly higher reward in relation with all other professions with the exception of psychologists/psychotherapists (M = 18.30, SD = 3.35), and these with the exception of physicians.

### Prevalence of effort-reward imbalance ratio (ERI ratio >1) in dependence of gender and profession

In the total sample, the prevalence of effort-reward imbalance ratio (ERI ratio >1) was 50.9%. The proportion of women with ERI ratio >1 was significantly higher than among men (women: 51.8%, men: 47.8%, χ^2^(1) = 7.001, p = 0.008, Phi = 0.034). Women showed an odds ratio (OR) for ERI ratio >1 that was 1.175 (CI = 1.043–1.324) times higher in comparison with men.

**Fig 1:**
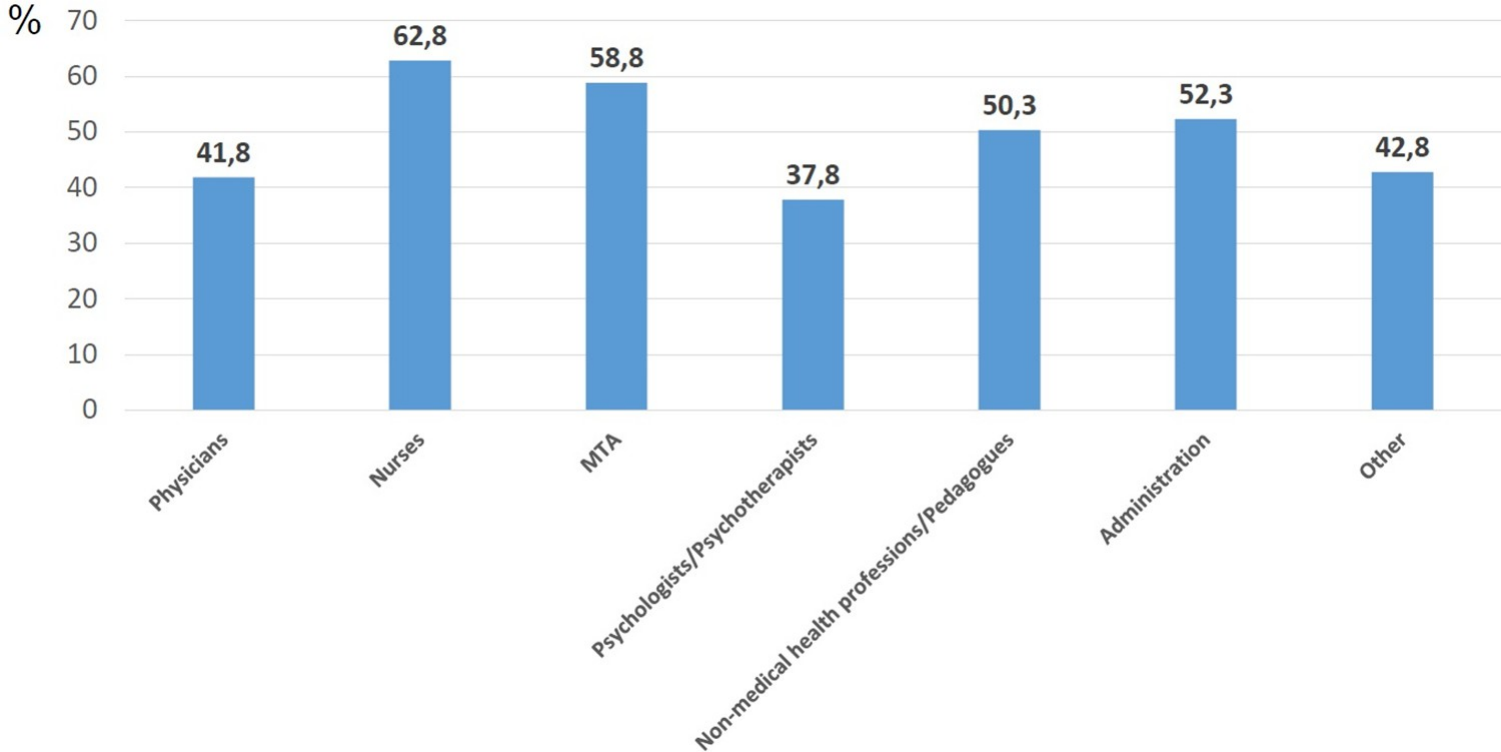
The prevalence of effort-reward imbalance ratio (ERI ratio > 1) in dependence of profession (N = 6174).

The occupational group was also significantly associated with the effort-reward imbalance ratio (χ^2^(1) = 182.869, p<0.001, Phi = 0.172). The highest prevalence of ERI imbalance was found among nurses (62.8%), followed by MTA (58.8%) (Fig 1). Psychologists/ psychotherapists revealed the lowest prevalence for ERI ratio >1 (37.8%), followed by physicians (41.8%) (Fig 1). A significantly higher proportion of nurses and MTA reported an ERI ratio >1 when compared with all other HCW. The proportion of HCW with ERI ratio >1 was significantly lower among psychologists/ psychotherapists and physicians in relation to all other groups.

In comparison with physicians, nurses were more than twice as likely (OR = 2.357, CI = 1.995–2.785) to show an ERI ratio >1, MTA (OR = 1.993, CI = 1.728–2.299), nonmedical therapists (OR = 1.412, CI = 1.200–1.662), administration (OR = 1.529, CI = 1.211–1.931) and psychologists/ psychotherapists (OR = 0.848, CI = 0.672–1.071).

### Prevalence of effort-reward imbalance ratio (ERI ratio >1) in dependence of other variables of interest

Table 1 shows the proportion of ERI ratio >1 in relation to socio-demographic and occupational characteristics and Table 2 in dependence of COVID-19-related characteristics and problems as well as working conditions. Most essential differences were observed regarding the items sufficient personnel for the current work load, sufficient recovery in free time and feeling protected by measures of the hospital/ the employer. HCW who disagreed to these three items achieved almost twice as frequently ERI ratio >1 than those who agreed.

### Correlates of ERI-effort, ERI-reward and ERI ratio

Most substantial correlations were found between increased efforts with insufficient personnel for the current work load (r = -0.431, p<0.001) and with insufficient recovery (r = -0.356, p<0.001) (Table 4).

**Table 4:** Correlations between ERI-effort and ERI-reward levels and effort-reward imbalance ratio (as continuous variable) with working conditions. Potential problems in the COVID-19 pandemic. Psychosocial resources and change in distress in the total sample (N = 6174).

		1	2	3	4	5	6	7	8	9	10	11	12	13	14
1. ERI_Effort	r	1	-0.278	0.800	-0.431	-0.356	-0.181	0.124	0.145	-0.110	-0.161	-0.089	-0.132	-0.009	0.147
	p		**<0.001**	**<0.001**	**<0.001**	**<0.001**	**<0.001**	**<0.001**	**<0.001**	**<0.001**	**<0.001**	**<0.001**	**<0.001**	0.466	**<0.001**
2. ERI_Reward	r		1	-0.727	0.255	0.301	0.344	-0.204	-0.220	0.266	0.419	0.215	0.273	0.171	-0.120
	p			**<0.001**	**<0.001**	**<0.001**	**<0.001**	**<0.001**	**<0.001**	**<0.001**	**<0.001**	**<0.001**	**<0.001**	**<0.001**	**<0.001**
3. ERI_Ratio	r			1	-0.423	-0.395	-0.311	0.198	0.210	-0.220	-0.345	-0.175	-0.238	-0.090	0.164
	p				**<0.001**	**<0.001**	**<0.001**	**<0.001**	**<0.001**	**<0.001**	**<0.001**	**<0.001**	**<0.001**	**<0.001**	**<0.001**
4. Sufficient staff	r				1	0.337	0.235	-0.089	-0.112	0.138	0.232	0.086	0.129	0.047	-0.121
	p					**<0.001**	**<0.001**	**<0.001**	**<0.001**	**<0.001**	**<0.001**	**<0.001**	**<0.001**	**<0.001**	**<0.001**
5. Sufficient recovery	r					1	0.279	-0.143	-0.169	0.236	0.218	0.215	0.315	0.134	-0.251
	p						**<0.001**	**<0.001**	**<0.001**	**<0.001**	**<0.001**	**<0.001**	**<0.001**	**<0.001**	**<0.001**
6. Trust in colleagues	r						1	-0.099	-0.068	0.158	0.298	0.216	0.195	0.147	-0.067
	p							**<0.001**	**<0.001**	**<0.001**	**<0.001**	**<0.001**	**<0.001**	**<0.001**	**<0.001**
7. Fear of becoming infected	r							1	0.632	-0.119	-0.225	-0.073	-0.229	-0.092	0.161
	p								**<0.001**	**<0.001**	**<0.001**	**<0.001**	**<0.001**	**<0.001**	**<0.001**
8. Fear to infect family	r								1	-0.106	-0.188	-0.033	-0.214	-0.079	0.129
	p									**<0.001**	**<0.001**	**0.010**	**<0.001**	**<0.001**	**<0.001**
9. Protection by national/ local authorities	r									1	0.414	0.140	0.189	0.097	-0.121
	p										**<0.001**	**<0.001**	**<0.001**	**<0.001**	**<0.001**
10. Protection by hospital/ employer	r										1	0.146	0.210	0.123	-0.078
	p											**<0.001**	**<0.001**	**<0.001**	**<0.001**
11. ESSI_sum	r											1	0.371	0.264	-0.014
	p												**<0.001**	**<0.001**	0.268
12. SOC_sum	r												1	0.437	-0.135
	p													**<0.001**	**<0.001**
13. Optimism	r													1	0.025
	p														**0.046**
14. Change_in_ distress	r														1
	p														

ERI = Effort-reward imbalance questionnaire; ESSI = ENRICHD Social Support Inventory; SOC = Sense of coherence; r = correlation coefficient; p = p value; significant p values are marked in bold.

Higher rewards were most essentially correlated with perceived protection by measures of the hospital/ the employer (r = 0.419, p<0.001), more trust in colleagues (r = 0.344, p<0.001) and sufficient recovery in free time (r = 0.301, p<0.001).

Regarding the ERI ratio as continuous variable, the highest correlation coefficients were observed between increased ERI ratio and insufficient personnel for the current work load (r = -0.423, p<0.001), insufficient recovery (r = -0.395, p<0.001) and unsatisfactory protection by measures of the hospital/ the employer (r = -0.345, p<0.001).

### Variables associated with ERI ratio >1

To analyze the associations of various factors of interest with the effort-reward imbalance ratio as criterion variable linear regression analyses were performed for the total sample (Table 5) and for women (S1 File) and men separately (S2 File).

**Table 5:** Multiple linear regression analysis for the effort-reward imbalance ratio as criterion variable in the total sample (N = 6174).

		Unstandardized Coefficients		Standardized Coeff.	t	p-value	95.0% CI for B		Collinearity Statistics	
		b	Std. Error	Beta			Lower Bound	Upper Bound	Tolerance	VIF
	Constant	1.735	0.048		35.866	**<0.001**	1.640	1.830		
	Gender (men vs. women)	-0.027	0.013	-0.023	-2.095	**0.036**	-0.052	-0.002	0.829	1.207
	Age+ (<41 vs. ≥ 41 years)	0.053	0.012	0.054	4.401	**<0.001**	0.030	0.077	0.682	1.467
	Living alone (no vs. yes)	-0.017	0.013	-0.015	-1.292	0.197	-0.043	0.009	0.807	1.239
	Having children (no vs. yes)	-0.016	0.013	-0.016	-1.273	0.203	-0.040	0.009	0.629	1.589
	Caring for old/sick relatives (no vs. yes)	0.006	0.013	0.005	0.452	0.651	-0.020	0.032	0.924	1.082
	Migration background (no vs. yes)	-0.036	0.016	-0.022	-2.195	**0.028**	-0.068	-0.004	0.981	1.019
	Employment (part-time vs. full-time)	0.037	0.011	0.038	3.283	**0.001**	0.015	0.060	0.779	1.283
	Working in patient care (no vs. yes)	0.029	0.015	0.022	1.929	0.054	0.000	0.059	0.758	1.319
	Physicians vs. nurses	0.134	0.017	0.098	7.846	**<0.001**	0.100	0.167	0.658	1.521
	Physicians vs. MTA	0.083	0.015	0.073	5.369	**<0.001**	0.053	0.113	0.546	1.832
	Physicians vs. psychologists/ psychotherapists	-0.005	0.023	-0.003	-0.232	0.817	-0.051	0.041	0.779	1.284
	Physicians vs. non-medical health professions	0.118	0.017	0.087	6.794	**<0.001**	0.084	0.152	0.627	1.596
	Physicians vs. administration staff	0.073	0.025	0.034	2.950	**0.003**	0.025	0.122	0.747	1.338
	Physicians vs. others	-0.016	0.021	-0.009	-0.781	0.435	-0.057	0.024	0.756	1.323
	Contact with infected patients (no vs. yes)	-0.023	0.014	-0.023	-1.651	0.099	-0.051	0.004	0.513	1.948
	Contact with contaminated material (no vs. yes)	0.092	0.014	0.092	6.528	**<0.001**	0.064	0.119	0.518	1.932
	Risk group due to preexisting illness (no vs. yes)	0.046	0.013	0.037	3.534	**<0.001**	0.020	0.071	0.930	1.076
	Occupancy of the wards (low/ average vs. high)	0.144	0.012	0.129	11.772	**<0.001**	0.120	0.168	0.852	1.174
	Home office (no vs. yes)	-0.014	0.013	-0.012	-1.095	0.273	-0.039	0.011	0.860	1.163
	Change of the department (no vs. yes)	0.025	0.014	0.018	1.728	0.084	-0.003	0.053	0.958	1.043
	Sufficient staff#	-0.081	0.004	-0.218	-18.793	**<0.001**	-0.089	-0.072	0.755	1.324
	Sufficient recovery#	-0.067	0.005	-0.177	-14.741	**<0.001**	-0.076	-0.058	0.712	1.405
	Trust in colleagues#	-0.058	0.005	-0.125	-11.210	**<0.001**	-0.068	-0.048	0.817	1.223
	Fear of becoming infected#	0.008	0.005	0.021	1.578	0.115	-0.002	0.019	0.556	1.798
	Fear to infect family#	0.021	0.005	0.059	4.327	**<0.001**	0.012	0.031	0.551	1.816
	Protection by national/ local authorities#	-0.009	0.005	-0.019	-1.690	0.091	-0.019	0.001	0.775	1.290
	Protection by hospital/ employer#	-0.065	0.005	-0.158	-13.181	**<0.001**	-0.075	-0.055	0.712	1.405
	Change in distress†	0.011	0.004	0.027	2.505	**0.012**	0.002	0.019	0.872	1.147
	Social support (sum score)	-0.004	0.001	-0.035	-2.958	**0.003**	-0.007	-0.001	0.739	1.353
	Sence of coherence (sum score)	-0.009	0.002	-0.066	-5.226	**<0.001**	-0.012	-0.005	0.632	1.582
	Optimism	0.009	0.004	0.023	2.050	**0.040**	0.000	0.017	0.780	1.283

F(31.6128) = 118.385. p<0.001; R^2^ = 0.375; adjusted R^2^ = 0.371; CI = confidence interval; + 0 = men. 1 = women; # 0 = strongly disagree. 1 = rather disagree. 2 = neither agree nor disagree. 3 = rather agree. 4 = strongly agree; † difference score in subjective burden: during the pandemic–before the pandemic (retrospective); MTA = medical technical assistants; significant p values are marked in bold.

Little´s MCAR test: for ENRICHD Social Support Inventory (ESSI): χ^2^ = 5.279, df = 3, p = 0.152; for Sense of Coherence Scale-3 (SOC-3): χ^2^ = 2.109, df = 2, p = 0.348; for effort-reward imbalance questionnaire (ERI), subscale reward: χ^2^ = 8.817, df = 12, p = 0.718.

For the total sample (Table 5), the “proportion of explained variance was 37.5%. Most essential observed factors (significantly and clinically relevant variables: standardized β≥0.1, independent of the sign) for an increased effort-reward imbalance ratio were: insufficient staff for the current work load (β = -0.218, p<0.001), insufficient recovery (β = -0.177, p<0.001), feeling insufficiently protected by measures taken by the hospital/the employer (β = -0.158, p<0.001), high occupancy rate of the wards (β = 0.129, p<0.001), insufficient trust in colleagues (β = -0.125, p<0.001) and being a nurse as compared with being a physician (β = 0.098, p<0.001). Other significant but less relevant factors can be found in Table 5.

In the regression model performed separately for women (explained variance: 36.5%) (S1 File), the most influential independent variables were the same as in the total sample: insufficient staff for the current work load (β = -0.219, p<0.001), insufficient recovery (β = -0.163, p<0.001), feeling insufficiently protected by measures taken by the hospital/the employer (β = -0.161, p<0.001), high occupancy rate of the wards (β = 0.134, p<0.001), insufficient trust in colleagues (β = -0.218, p<0.001) and being a nurse as compared with being a physician (β = 0.092, p<0.001).

In male HCW (explained variance: 41.8%) (S2 File) increased ERI ratio was substantially associated with: insufficient recovery (β = -0.222, p<0.001), insufficient staff for the current work load (β = -0.219, p<0.001), feeling insufficiently protected by measures taken by the hospital/the employer (β = -0.149, p<0.001), insufficient trust in colleagues (β = -0.120, p<0.001), being a nurse (in comparison with being a physician) (β = 0.115, p<0.001) and high occupancy rate of the wards (β = -0.112, p<0.001).

## Discussion

The aim of the current study was to assess the level of work stress and occupational recognition, as well as the potential resulting an effort-reward imbalance among German HCW during the first wave of the COVID-19 pandemic. In particular, the type of health profession and gender should be considered to uncover potential differences. In addition, the aim was to ascertain whether further factors had an influence on experienced effort and reward. The focus here was on the work demands for HCWs that occurred during the first wave of the COVID-19 pandemic.

Since the COVID-19 outbreak more than two years ago, healthcare systems worldwide have been severely challenged. We know from Asian studies in particular [9–12] that this has implications for the physical and mental health of HCW. Scores for depression, anxiety, or fatigue increased significantly at pandemic times. HCW generally have high workloads, which are also associated with rather low social and monetary reward in some occupational fields. Under pandemic conditions, these conditions are again significantly exacerbated [49, 50].

Because the EgePan Unimed study was not developed until the outbreak of the pandemic, there is no survey data for the underlying sample from the items used prior to the onset of the pandemic. This was captured retrospectively by two questions. HCW had to face a virus posed a potential threat to their own integrity and that of their family and friends. In our study, 40% of the respondents had contact with COVID patients or with contaminated material. Just under half of the HCW respondents reported fear of infecting their relatives. The fear of infecting themselves was significantly lower. Since the actual risk of becoming infected with the Corona virus can be classified as very high, this is a realistic fear. COVID 19 can be transmitted before the carrier himself shows symptoms and is therefore never sure to maybe infect further contacts. Since clinics and medical care institutions generally have particularly good hygiene concepts for direct patient contact and dealing with COVID patients may have become the norm in some places, a strong decrease in fears could potentially be observed. Previous studies during the Ebola epidemic showed that the fear of infection can have an influence on work motivation and the handling of the disease or with the treatment [51, 52]. Therefore, the protective measures of the employer are of particular importance: at the time of the survey, only just under half (47.8%) felt sufficiently protected by the employer measures, even less by those of the local and national authorities. Improvements are therefore urgently needed in this context.

Siegrist’s ERI questionnaire was used to assess the associations between (pandemic) working conditions and psychological effects [16]. The analyses showed that although no gender differences were found for the effort scale, differences were found for the reward scale. Thus, it can be concluded that although men and women in this study rated their effort as similar, men felt significantly more rewarded. However, the effect size was very small. In relation to the different occupational groups, nurses and administrative staff report significantly higher effort than other occupational groups, and psychologists and psychotherapists rated their effort significantly lower than for all other occupational groups. However, together with the group of physicians, these receive significantly higher reward, while nurses and MTAs report significantly lower values here than all other occupational groups. Several aspects can be discussed here to explain the results. First, a university degree seems to be associated with significantly higher values of reward than an apprenticeship [53]. Another aspect is the controllability of the job, referred to by Siegrist as "job control," that may seem to play a role [54, 55]. The framework of psychologists and psychotherapists is more predefined than that of a nurse, for whom it is often completely unclear at admission how care-intensive a patient is. Overall, it is the case that social professions are often held in lower esteem. Elevated ERI values are observed in more than 50% of the sample. Women, nurses and MTAs have particularly high ERI-ratios, although for gender it is fed exclusively by differences on the "recognition" scale.

Correlation analyses show expected directions. Recognition correlates positively with psychosocial resources, lower anxiety or a higher sense of protection, more time for recreation and better working conditions. On the other hand an opposite correlation is found for the effort scale.

In order to identify the factors that significantly account for the imbalance and to learn more about the nature of the correlation of the variables from the correlation analysis, a linear regression analysis was performed. This was able to explain 37.5% of the variance in the final model and provide important findings as to where changes and improvements could be made. In addition to personality factors such as sense of coherence, social support was recorded as a protective factor. However, it must also be mentioned that even before the outbreak of the pandemic, the occupational groups surveyed were considered "vulnerable" to an imbalance between effort and reward according to Siegrist’s ERI model [56, 57]. Numerous studies indicate that social status, salary, shift work, overtime, dealing with serious illness and death, and low staffing levels contribute significantly to an unfavorable ERI ratio [43, 57]. The data of our study highlight the relevance of insufficient opportunity for regeneration, high numbers of patients on wards, wrong or untrustworthy staff, too few protective measures against infection accompanied by fear of infection. This is exactly where future measures should focus on. Obviously, the pandemic events have a reinforcing effect on the increasing workload compression that has already existed for some time, the structural and personnel bottlenecks, and even subjectively perceived social inequality of the medical professional contexts.

One factor that continues to receive rather step-motherly treatment is the influence of gender. In our survey, approximately 77% of participants were female as well as most nurses were female. With flexibility, high patient demand, and shift work it is quite difficult to obtain a compatibility of work and family as care work that is still primarily women’s. Research indicates that men care significantly more about job reward and advancement opportunities than women [58–60]. The latter choose professions in the social sector more often than men. The question is: are women more likely to choose occupations with less or little reward, or do social occupations have so little reward, because that is where so many women work? Future studies should focus more specifically on the gender factor in order to be able to implement targeted measures here.

### Strengths and limitations

To the best of our knowledge, this study is the largest survey on mental health of HCW and its associations with effort-reward imbalance during the first wave of the COVID-19 pandemic in Europe. Nevertheless, our study also has several limitations that must be considered when interpreting the results. Due to the cross-sectional design causal conclusions for the measured variables cannot be drawn. However, the VOICE survey consists of four measurement points. Thus, further publications will analyze the prospective findings. Another limitation concerns the recruitment method. The present study was a voluntary online survey with recruitment primarily in selected university, other hospitals and through professional societies, social media and word of mouth. Therefore, a selection bias cannot be excluded. In addition, the present study is subject to potential response bias, meaning that too burdened or less burdened persons did not participate. The composition of the sample, which includes a significantly higher proportion of women, is not representative of all occupational groups. Therefore, the findings cannot be regarded as representative for all HCW in Germany. In addition, it should be mentioned that no specific and controllable target group was addressed in the recruitment, except for the 4 university hospitals. Therefore, it is not possible to draw conclusions about the response rates of the individual professional groups from different sectors. The response rates shown for university hospitals were very low, with a maximum of 24.5% (MTAs). This is undoubtedly a limitation that raises the question of generalizing the results to the professional groups working in the fields. A possible explanation for this could be selection effects. It might be that people who felt more stressed felt more strongly affected by the study and its participation. Due to the large sample size and the descriptive and partly exploratory nature of the results presented, these and the interpretations given should be understood as initial possibilities and suggestions for further research.

## Conclusions

Our results indicate a high proportion of HCW with effort-reward imbalance, while there are substantial profession-related differences. Preventive interventions should be offered to vulnerable groups among the HCW to decrease the imbalance. According to the data of our study, nurses and MTAs deserve special attention [61–63]. Regarding MTAs in particular, there is currently little research outside our own study group that draws attention to the high imbalance of their work. In order to make the still ongoing increased workload bearable for HCW, it seems necessary to make changes in the areas of recognition, social support, staffing ratios, recovery times, collegial cooperation, and hygiene measures in the interest of HCW.

Our study also detected significant gender differences concerning reward. Future research should explore in detail which mechanisms could be responsible for this gender gap and appropriate measures at political level should be taken to eliminate observed inequality. This is an important issue for the health care system because an effort-reward imbalance has been shown to lead to increased sick leave days and turnover intention e.g. among nurses [64].

Finally, a variety of factors concerning unfavorable working conditions or resulting from these such as insufficient staff for the current workload, insufficient recovery, high occupancy rate of the wards, insufficient perceived protection by measures taken by the hospital/the employer were associated with increased effort-reward imbalance, while social support, SOC and trust in colleagues were related to a decreased risk. Therefore, improving the working conditions of HCW and strengthening their individual resources and the team spirit would have a positive influence on the perceived effort-reward ratio.

## Supporting information

S1 File Multiple linear regression analysis for the effort-reward imbalance ratio as criterion variable for female health care workers. (DOCX)

S2 File Multiple linear regression analysis for the effort-reward imbalance ratio as criterion variable for male health care workers. (DOCX)
