# Supplementary material for: The prevalence of effort-reward imbalance and its associations with working conditions, psychosocial resources and burden among health care workers during the COVID-19 pandemic: Results of the egePan-Voice study
Source: PLoS One. 2023 Aug 17;18(8):e0287509. doi: 10.1371/journal.pone.0287509 (PMC10434884; doi:10.1371/journal.pone.0287509)
Supplement: S1 File — (DOCX) [file pone.0287509.s001.docx]

| **SUPPLEMENTUM 1.** **Multiple linear regression analysis for the effort-reward imbalance ratio as criterion variable for female health care workers.** | | | | | | | | | | |
| --- | --- | --- | --- | --- | --- | --- | --- | --- | --- | --- |
|  | | Unstandardized Coefficients | | Standardized Coeff. | t | Sig. | 95.0% CI for B | | Collinearity Statistics | |
|  |  | B | Std. Error | Beta |  |  | Lower Bound | Upper Bound | Tolerance | VIF |
|  | (Constant) | 1.676 | 0.054 |  | 31.110 | **<0.001** | 1.571 | 1.782 |  |  |
|  | Age+ (<41 vs. ≥ 41 years) | 0.059 | 0.014 | 0.060 | 4.287 | **<0.001** | 0.032 | 0.086 | 0.678 | 1.474 |
|  | Living alone (no vs. yes) | -0.013 | 0.015 | -0.011 | -0.892 | 0.373 | -0.042 | 0.016 | 0.821 | 1.217 |
|  | Having children (no vs. yes) | -0.010 | 0.014 | -0.011 | -0.725 | 0.468 | -0.039 | 0.018 | 0.611 | 1.638 |
|  | Caring for old/sick relatives (no vs. yes) | <0.001 | 0.015 | 0.000 | -0.008 | 0.993 | -0.029 | 0.029 | 0.915 | 1.093 |
|  | Migration background (no vs. yes) | -0.035 | 0.018 | -0.022 | -1.920 | 0.055 | -0.071 | 0.001 | 0.978 | 1.022 |
|  | Employment (part-time vs. full-time) | 0.037 | 0.013 | 0.039 | 2.951 | **0.003** | 0.013 | 0.062 | 0.781 | 1.281 |
|  | Working in patient care (no vs. yes) | 0.029 | 0.017 | 0.023 | 1.753 | 0.080 | -0.003 | 0.062 | 0.779 | 1.284 |
|  | Physicians vs. nurses | 0.124 | 0.020 | 0.092 | 6.209 | **<0.001** | 0.085 | 0.163 | 0.615 | 1.627 |
|  | Physicians vs. MTA | 0.079 | 0.018 | 0.074 | 4.490 | **<0.001** | 0.045 | 0.113 | 0.498 | 2.010 |
|  | Physicians vs. psychologists/ psychotherapists | -0.017 | 0.026 | -0.009 | -0.652 | 0.514 | -0.069 | 0.034 | 0.738 | 1.355 |
|  | Physicians vs. non-medical health professions | 0.111 | 0.019 | 0.089 | 5.790 | **<0.001** | 0.073 | 0.149 | 0.572 | 1.750 |
|  | Physicians vs. administration staff | 0.067 | 0.028 | 0.032 | 2.391 | **0.017** | 0.012 | 0.123 | 0.739 | 1.353 |
|  | Physicians vs. others | -0.025 | 0.026 | -0.013 | -0.984 | 0.325 | -0.075 | 0.025 | 0.745 | 1.342 |
|  | Contact with infected patients (no vs. yes) | -0.031 | 0.016 | -0.031 | -1.962 | **0.050** | -0.062 | 0.000 | 0.547 | 1.829 |
|  | Contact with contaminated material (no vs. yes) | 0.099 | 0.016 | 0.098 | 6.247 | **<0.001** | 0.068 | 0.130 | 0.540 | 1.853 |
|  | Risk group due to preexisting illness (no vs. yes) | 0.064 | 0.015 | 0.052 | 4.353 | **<0.001** | 0.035 | 0.092 | 0.927 | 1.078 |
|  | Occupancy of the wards (low/ average vs. high) | 0.149 | 0.014 | 0.134 | 10.713 | **<0.001** | 0.122 | 0.176 | 0.852 | 1.174 |
|  | Homeoffice (no vs. yes) | -0.008 | 0.015 | -0.007 | -0.574 | 0.566 | -0.037 | 0.020 | 0.855 | 1.170 |
|  | Change of the department (no vs. yes) | 0.024 | 0.017 | 0.017 | 1.455 | 0.146 | -0.008 | 0.057 | 0.964 | 1.037 |
|  | Sufficient staff# | -0.080 | 0.005 | -0.219 | -16.483 | **<0.001** | -0.089 | -0.070 | 0.760 | 1.315 |
|  | Sufficient recovery# | -0.062 | 0.005 | -0.163 | -11.900 | **<0.001** | -0.072 | -0.052 | 0.713 | 1.402 |
|  | Trust in colleagues# | -0.058 | 0.006 | -0.127 | -9.940 | **<0.001** | -0.070 | -0.047 | 0.824 | 1.214 |
|  | Fear of becoming infected# | 0.007 | 0.006 | 0.017 | 1.118 | 0.264 | -0.005 | 0.018 | 0.570 | 1.754 |
|  | Fear to infect family# | 0.026 | 0.006 | 0.071 | 4.647 | **<0.001** | 0.015 | 0.036 | 0.571 | 1.750 |
|  | Protection by national/ local authorities# | -0.004 | 0.006 | -0.010 | -0.744 | 0.457 | -0.016 | 0.007 | 0.764 | 1.309 |
|  | Protection by hospital/ employer# | -0.067 | 0.006 | -0.161 | -11.685 | **<0.001** | -0.078 | -0.055 | 0.703 | 1.422 |
|  | Change in distress† | 0.005 | 0.005 | 0.014 | 1.099 | 0.272 | -0.004 | 0.015 | 0.870 | 1.150 |
|  | Social support (sum score) | -0.004 | 0.002 | -0.035 | -2.571 | **0.010** | -0.008 | -0.001 | 0.744 | 1.344 |
|  | Sence of coherence (sum score) | -0.008 | 0.002 | -0.063 | -4.353 | **<0.001** | -0.012 | -0.005 | 0.636 | 1.571 |
|  | Optimism | 0.008 | 0.005 | 0.021 | 1.581 | 0.114 | -0.002 | 0.017 | 0.777 | 1.288 |

F(30.4733)=90.877. p<0.001; R^2^=0.365; adjusted R^2^=0.361; CI = confidence interval; VIF = variance inflation factor; # 0 = strongly disagree. 1 = rather

disagree. 2 = neither agree nor disagree. 3 = rather agree. 4 = strongly agree; † difference score in subjective burden: during the pandemic – before the pandemic (retrospective); MTA = medical technical assistants; significant p values are marked in bold.
