# Supplementary material for: The prevalence of effort-reward imbalance and its associations with working conditions, psychosocial resources and burden among health care workers during the COVID-19 pandemic: Results of the egePan-Voice study
Source: PLoS One. 2023 Aug 17;18(8):e0287509. doi: 10.1371/journal.pone.0287509 (PMC10434884; doi:10.1371/journal.pone.0287509)
Supplement: S2 File — (DOCX) [file pone.0287509.s002.docx]

| **SUPPLEMENTUM 2.** **Multiple linear regression analysis for the effort-reward imbalance ratio as criterion variable for male health care workers.** | | | | | | | | | | |
| --- | --- | --- | --- | --- | --- | --- | --- | --- | --- | --- |
|  | | Unstandardized Coefficients | | Standardi-zed Coeff. | t | Sig. | 95.0% CI for B | | Collinearity Statistics | |
|  |  | B | Std. Error | Beta |  |  | Lower Bound | Upper Bound | Tolerance | VIF |
|  | (Constant) | 1.869 | 0.104 |  | 17.953 | **<0.001** | 1.665 | 2.074 |  |  |
|  | Age+ (<41 vs. ≥ 41 years) | 0.020 | 0.026 | 0.020 | 0.780 | 0.436 | -0.031 | 0.072 | 0.664 | 1.507 |
|  | Living alone (no vs. yes) | -0.052 | 0.031 | -0.040 | -1.648 | 0.100 | -0.113 | 0.010 | 0.724 | 1.381 |
|  | Having children (no vs. yes) | -0.031 | 0.027 | -0.030 | -1.144 | 0.253 | -0.084 | 0.022 | 0.638 | 1.568 |
|  | Caring for old/sick relatives (no vs. yes) | 0.024 | 0.031 | 0.017 | 0.781 | 0.435 | -0.036 | 0.084 | 0.946 | 1.057 |
|  | Migration background (no vs. yes) | -0.038 | 0.036 | -0.023 | -1.077 | 0.282 | -0.108 | 0.031 | 0.965 | 1.037 |
|  | Employment (part-time vs. full-time) | 0.048 | 0.030 | 0.034 | 1.593 | 0.111 | -0.011 | 0.107 | 0.914 | 1.094 |
|  | Working in patient care (no vs. yes) | 0.021 | 0.038 | 0.014 | 0.540 | 0.589 | -0.054 | 0.096 | 0.624 | 1.603 |
|  | Physicians vs. nurses | 0.163 | 0.034 | 0.115 | 4.840 | **<0.001** | 0.097 | 0.229 | 0.752 | 1.330 |
|  | Physicians vs. MTA | 0.073 | 0.035 | 0.049 | 2.056 | **0.040** | 0.003 | 0.143 | 0.741 | 1.349 |
|  | Physicians vs. psychologists/ psychotherapists | 0.051 | 0.056 | 0.020 | 0.917 | 0.359 | -0.058 | 0.160 | 0.879 | 1.138 |
|  | Physicians vs. non-medical health professions | 0.130 | 0.053 | 0.053 | 2.445 | **0.015** | 0.026 | 0.234 | 0.900 | 1.111 |
|  | Physicians vs. administration staff | 0.077 | 0.056 | 0.035 | 1.393 | 0.164 | -0.032 | 0.187 | 0.680 | 1.472 |
|  | Physicians vs. others | -0.010 | 0.036 | -0.007 | -0.282 | 0.778 | -0.081 | 0.061 | 0.751 | 1.331 |
|  | Contact with infected patients (no vs. yes) | 0.010 | 0.032 | 0.010 | 0.296 | 0.767 | -0.054 | 0.073 | 0.411 | 2.431 |
|  | Contact with contaminated material (no vs. yes) | 0.055 | 0.032 | 0.054 | 1.706 | 0.088 | -0.008 | 0.118 | 0.423 | 2.365 |
|  | Risk group due to preexisting illness (no vs. yes) | -0.015 | 0.027 | -0.012 | -0.553 | 0.581 | -0.069 | 0.039 | 0.919 | 1.088 |
|  | Occupancy of the wards (low/ average vs. high) | 0.128 | 0.026 | 0.112 | 4.986 | **<0.001** | 0.078 | 0.178 | 0.840 | 1.190 |
|  | Homeoffice (no vs. yes) | -0.031 | 0.027 | -0.026 | -1.170 | 0.242 | -0.083 | 0.021 | 0.853 | 1.172 |
|  | Change of the department (no vs. yes) | 0.019 | 0.030 | 0.014 | 0.652 | 0.515 | -0.039 | 0.078 | 0.916 | 1.092 |
|  | Sufficient staff# | -0.083 | 0.009 | -0.219 | -8.993 | **<0.001** | -0.102 | -0.065 | 0.719 | 1.390 |
|  | Sufficient recovery# | -0.087 | 0.010 | -0.222 | -8.912 | **<0.001** | -0.106 | -0.068 | 0.685 | 1.459 |
|  | Trust in colleagues# | -0.058 | 0.011 | -0.120 | -5.150 | **<0.001** | -0.081 | -0.036 | 0.784 | 1.276 |
|  | Fear of becoming infected# | 0.016 | 0.012 | 0.040 | 1.386 | 0.166 | -0.007 | 0.039 | 0.503 | 1.988 |
|  | Fear to infect family# | 0.003 | 0.011 | 0.008 | 0.267 | 0.789 | -0.018 | 0.024 | 0.489 | 2.043 |
|  | Protection by national/ local authorities# | -0.021 | 0.010 | -0.048 | -2.082 | **0.038** | -0.042 | -0.001 | 0.788 | 1.269 |
|  | Protection by hospital/ employer# | -0.062 | 0.010 | -0.149 | -6.141 | **<0.001** | -0.082 | -0.042 | 0.722 | 1.385 |
|  | Change in distress† | 0.031 | 0.009 | 0.075 | 3.395 | **<0.001** | 0.013 | 0.049 | 0.863 | 1.159 |
|  | Social support (sum score) | -0.004 | 0.003 | -0.033 | -1.323 | 0.186 | -0.010 | 0.002 | 0.704 | 1.419 |
|  | Sence of coherence (sum score) | -0.011 | 0.004 | -0.078 | -2.949 | **0.003** | -0.018 | -0.004 | 0.615 | 1.627 |
|  | Optimism | 0.013 | 0.009 | 0.034 | 1.459 | 0.145 | -0.004 | 0.030 | 0.767 | 1.304 |

F(30.1365)=32.646. p<0.001; R^2^=0.418; adjusted R^2^=0.405; CI = confidence interval; VIF = variance inflation factor; # 0 = strongly disagree. 1 = rather disagree.

2 = neither agree nor disagree. 3 = rather agree. 4 = strongly agree; † difference score in subjective burden: during the pandemic – before the pandemic

(retrospective); MTA = medical technical assistants; significant p values are marked in bold.
